# Development and Psychometric Validation of the Lincoln Canine Anxiety Scale

**DOI:** 10.3389/fvets.2020.00171

**Published:** 2020-04-03

**Authors:** Daniel S. Mills, Hanns Walter Mueller, Kevin McPeake, Odilo Engel

**Affiliations:** ^1^Animal Behaviour Cognition and Welfare Group, School of Life Sciences, University of Lincoln, Lincoln, United Kingdom; ^2^Boehringer Ingelheim Pharma GmbH und Co KG, Ingelheim am Rhein, Germany; ^3^Boehringer Ingelheim Vetmedica GmbH, Ingelheim am Rhein, Germany

**Keywords:** anxiety, dog, fear, phobia, scale, validation

## Abstract

**Introduction:** Anxiety in dogs, especially in relation to certain noises, is a common issue which can lead to clinically significant problems like noise phobias. While several scales have been used to assess sound sensitivity and reactivity, clinical monitoring has tended to depend on unvalidated methods, general assessment, and/or historical comparison with owners' recall of previous episodes. Therefore, we aimed to develop and validate a scale to assess canine anxiety.

**Materials and Methods:** We used the data from 226 dogs from a previously reported double blind placebo controlled study in order to determine the validity of the 16 item “Lincoln Canine Anxiety Scale.” Unidimensionality was assessed through correlation between individual item scores and total score, with internal consistency assessed using Cronbach's alpha. Factor analysis was used to determine the dimensionality of the scale. Item response theory (IRT) was used to gain insight into the value of single items to the overall scale scores. To characterize the score characteristics in an anxiety-eliciting context we analyzed the behaviors of placebo treated dogs assessed at 00:20 h, the time point of maximum noise stimulus during New Year's Eve fireworks. Sensitivity of the scale to treatment effects was determined from its performance in the wider study.

**Results:** The majority of correlations between individual items and total score were >0.48, with Cronbach's alpha equalling 0.88, indicating good internal consistency. Principal Component Analysis (PCA) confirmed a unidimensional structure. IRT indicated that the scale could be reduced to 11 items without significantly reducing its value. The scale showed good treatment and stimulus sensitivity, with a score change of ~20 points differentiating “no/worse” effect from an “excellent” effect and a 30% difference between treatment (imepitoin) and placebo.

**Conclusion:** In our initial validation the Lincoln Canine Anxiety Scale appears to provide a reliable method for determining anxiety and fear responses by dogs and monitoring the effects of treatment.

## Introduction

Fear (the response to perceived threatening stimuli or scenarios in the present environment) and anxiety (the preparatory response made in anticipation of threatening stimuli or scenarios) are fundamental adaptive responses to, being evolutionary highly conserved (1). However, excessive anxiety and fear can become a behavior problem which impairs daily functioning for the affected individual and its immediate associates. The point where the behavior is excessive and enduring can be considered diagnostic of a clinical disorder (e.g., phobia) (2). Excessive anxiety underlies many behavior problems in dogs (3–5). However, the point “at which a fear becomes a phobia is unknown” (6), and to date no study has shown that the behavioral response of dogs becomes qualitatively different at any particular point. All data to date indicate that the behavioral response to an aversive event or situation is dimensional in nature, and thus exists along a spectrum which includes normality. The anxiety spectrum in dogs is marked by different clinical presentations (5), and its diagnostic categorization can include the eliciting context(s) e.g., firework fear. At the same time, there is a clinical need to characterize the severity of the signs, which is especially important within clinical trials as we seek to build the evidence base within veterinary behavioral medicine. This may determine appropriate patient profiles for an intervention or help establish the efficacy of a treatment.

Anxiety and fear related to anxiety disorders in people are usually assessed by rating scales, often referred to as some form of anxiety assessment instrument, such as the Hamilton Rating Scale for Anxiety (HAM-A) (7) or the Screen for Child Anxiety Related Emotional Disorders (SCARED) (8). While in adults self-report is the standard, the routine assessment by proxies (parents or other closely related persons) for children shows that reporting by others can be a valid way to assess these conditions (2). As dogs cannot verbally express their internal states, systematic observations of their behavior, and physiology (e.g., pupil dilation, panting) in relation to a given context are often used to infer underlying state (9). However, while such approaches may be useful for inferring the presence of a given emotional state, reliably quantifying severity represents a separate challenge.

Dimensional ratings scales and behavior tests are widely used in veterinary behavior research, however their reliability and construct validity is often not reported or not sufficiently examined (10). Validity is the extent to which a test measures what it is intended to measure, which can be especially challenging for subjective personal evaluations like an individual's perceived quality of life, anxiety, or pain (11), a challenge exacerbated further when the subject is non-verbal (e.g., a young child or non-human animal). A comparison to other independent and indisputable clinical signs is often not useful due to the nature of the measured concept. These concepts with no objective gold standard are commonly validated using a series of steps to build “construct validity.” This involves an assessment of the comprehensiveness of the scale (face validity); correlational evidence, which is often presented in association with factor analysis of the items making up the scale (concurrent validity); and evidence for the ability of the instrument to discriminate between different groups (predictive validity) (11). To date, there are very few validated clinical scales relating to behavior problems in dogs (12–14), and there is no validated general scale to assess the severity of their anxiety, fear, or related state. Therefore, we aimed to develop and validate a scale to assess canine anxiety.

## Methods

### Sample and Data Collection

**Figure 1:**
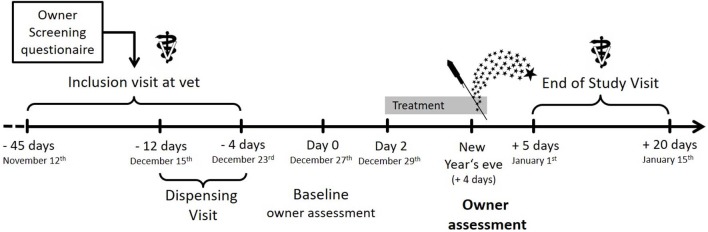
Study design of the clinical trial, where the dataset was used for this study Image from Engel et al. (15).

We used the dataset previously collected in a prospective, placebo-controlled, randomized, double-blinded, clinical trial (15) (Full Analysis Set following the Intention to Treat principle) in order to evaluate aspects of the quality of psychometric instrument used to evaluate treatment in this study (15). This clinical trial demonstrated the efficacy and safety of the partial low-affinity GABA_A_ agonist imepitoin in comparison to placebo for the control of anxiety and fear associated with noise phobia in privately owned dogs. The study design used a predictable and global noise event as eliciting context: the traditional New Year's Eve fireworks in Germany and the Netherlands (Figure 1).

All dogs in this study (15) were diagnosed with noise phobia based on medical history and the Lincoln Sound Sensitivity Scale (16, 17), and met the inclusion criteria of this trial. As the scale consists of 16 behaviors, a score of at least 30 was used to qualify for inclusion as it indicates that, at a theoretical level, at the very least, either every sign would have to occur at its lowest intensity (score 1) frequently (score 2) [1^*^2^*^16 signs = 32] or a minimum of two signs are occurring in their most intense form (score 5) every time the noise was heard (score 3) [5^*^3^*^2 signs =30] or a larger number of signs are occurring at a lower intensity but at least frequently. This captures the essence of the definition of noise phobia, i.e., that it is an “excessive fear of a sound,” since *excessive* can be considered to refer to either the severity of signs, or the number of signs (and the cut off used here would indicate that if signs are occurring at a lower intensity that there must be many more signs).

Four days before New Year's Eve and prior to treatment starting, the owners performed a baseline assessment using the “Lincoln Canine Anxiety Scale” (LCAS) scale. This is described as a questionnaire modified from the Lincoln Sound Sensitivity Scale in the original study (15). In keeping with the convention in the human literature, we refer to the instrument from now on as an anxiety scale, accepting that it may be assessing other emotional states. This terminological convention is used since it is the anticipation of danger which tends to drive whatever emotional state or disorder which ultimately presents as the problem. Two days before the anticipated noise event, treatment was started (i.e., December 29th) either with imepitoin 30 mg/kg body weight b.i.d. or placebo. On New Year's Eve, owners were asked to score their observations at fixed, representative time points (i.e., 16:00 h; 22:00 h; 0:20 h, and 1:00 h) on the anxiety scale (LCAS). In addition, the owners were asked to report their impression of the overall treatment effect the day after fireworks occurred. In both assessments a significant improvement was observed under imepitoin treatment compared to placebo.

### Construction of the Anxiety Score

The instrument consists of a 16-item owner-report measure of anxiety signs. The items in the “Lincoln Canine Anxiety Scale” (LCAS) are phrased in a closed format using a differential scale (Table 1).

**Table 1:** Design of the question responses for each of the 16 behaviors in the Lincoln Canine Anxiety Scale (LCAS).

Behavior	0 Not present 1 Indicator for Score 1 (e.g., small amount) 2 3 4 5 Indicator for Score 5 (e.g., extensive amount)

*The wording for the indicator for each behavior is presented in Table 2*.

It is based on the established and widely accepted content of the instrument used to determine noise phobia [Lincoln Sound-Sensitivity Scale) in the main clinical behavioral study (18)]. The states referred to in this instrument have been used in several clinical trials on noise phobia and other anxiety problems with success (19, 20), and also used in other assessments with a similar goal (21) and the Anxiety Intensity Rank scale (22). However, we did not include the behavior “Exaggerated response when startled,” from the Lincoln Sound-Sensitivity Scale, because it is rare and previous experience of one of the authors and originators of the scale (DSM,) indicate that it is difficult for the owners to judge. In addition, all text fields specifying specifics of a behavior were omitted. The Lincoln Sound-Sensitivity Scale has a focus on assessing anxiety/fear reactions retrospectively over an extended period of time and thus considers how often a specific behavior is displayed in response to a noise situation (frequency). In contrast, the LCAS was designed to assess the nature of a given behavior over a more limited fixed sampling point (i.e., 15 min). Therefore, only the intensity of the displayed behavior was rated by respondents (Table 2).

**Table 2:** Behaviors assessed in the Lincoln Canine Anxiety Scale (LCAS).

**Behavior**	**Indicator for Score 0**	**Indicator for Score 1**	**Indicator for Score 5**
Running around	Not present	Small amount—occasional burst of activity	Extensive amount—continuously running around
Drooling Saliva	Not present	Small amount—damp around mouth	Extensive amount—pools of saliva
Hiding (e.g., under furniture, behind owner, etc.)	Not present	Small amount—retreats work to get dog from hiding area	Extensive amount—will not be removed from hiding area
Destructiveness (e.g., furniture, doors, carpets, …)	Not present	Small amount—small item, e.g., pens	Extensive amount—e.g., holes in the wall
Cowering (e.g., tucks tail, flattens ears, etc.)	Not present	Small amount—uneasy	Extensive amount—petrified
Restlessness/Pacing	Not present	Small amount—occasional burst of activity	Extensive amount—fixed route continuously traced
Aggressive behavior (e.g., growling, snapping, or biting)	Not present	Small amount—occasional growl	Extensive amount—severe biting attempts made
“Freezing to the spot”	Not present	Occurs sporadically within an event	Most of the time
Barking/Whining/Howling	Not present	Small amount	Extensive amount
Panting	Not present	Small amount—occurs sporadically within an event	Most of the time
Vomiting, Defecating, Urinating, and/or Diarrhea	Not present	Small amount	Extensive amount
Owner seeking behavior	Not present	Seeks out owner occasionally during the event	Will not leave owner in any circumstance
Vigilance/Scanning of the environment	Not present	Occurs sporadically within an event	Most of the time
Bolts	Not present	Occurs occasionally, in response to certain noises	Occurs always, in response to a wide range of sounds
Shaking or trembling	Not present	Occurs occasionally, in response to certain noises	Occurs always, in response to a wide range of sounds
Self-harm	Not present	Small amount—e.g., licking feet	Extensive amount—e.g., broken teeth or nails

Owners were asked to fill in the questionnaire each time, to indicate the occurrence and intensity of the respective behavior in the observation period [the preceding 15 min per time point- see (15) for further details]. Behaviors that did not occur are scored as zero, and the severity of observed behaviors was scored on an ordinal scale from 1 (a small amount, behaviourally elaborated according to the specific item) to 5 (an extensive amount behaviourally elaborated). The assessment is a differential scale and asks where the respondent's position is between two bipolar options. These two extremes on the item score are easy to understand, and leaning toward one or the other extreme does not require any expertise in grading the response. In contrast, Likert style scales or “Choice of categories” ask for agreement to a certain category or question, and not for the impression or attitude of the respondent.

### Other Scores

The nature of a problem is such that, by definition, it involves a perception by someone (in this case the owner) of something (in this case their dog's behavior) that causes this some discomfort (the perceived problem); accordingly, the owner's subjective impression is an important part of the perceived magnitude of the problem and thus also treatment success. Hence the owners performed an overall assessment of the treatment effect on an ordinal scale to subjectively rate how well they thought the dog coped with the noise events (Table 3). Responses were given within a closed (forced choice) format from a “choice of categories.” As perceptions are usually graded from extreme positive to extreme negative, this 5-point ordinal rating scale was employed to cover a continuum of possible impressions from an “Excellent Effect” to “Worse Effect,” through “Some Effect,” “Good Effect,” and “No Effect.”

**Table 3:** Rating of owner's proxy assessment of overall treatment effects on the dog.

**Score**	**Description**
Excellent effect	The dog does not react to fireworks with anxious/fearful behavior at all
Good effect	The dog's reactions are mild and it can calm down
Some effect	The dog is reacting somewhat less/milder than in previous year(s) without treatment but it cannot calm down
No effect	There is no reduction/change in the dog's reactions compared to previous year(s) without treatment
Worse effect	The dog's reaction to fireworks is stronger than in previous year(s) without treatment

*In gray marked scores indicate an insufficient treatment response*.

### Data Analyses

Psychometric analysis validation is largely dependent on the statistical methods chosen. Where possible, we sought to use the largest dataset available covering the widest range of presentations of the problem, i.e., both imepitoin treatment and placebo groups in the study [see Sample and Data Collection and reference (15)], to describe the structure of LCAS. A pre-requisite of such an investigation is consideration of the demographic characteristics of the underlying study data to identify possible imbalances in the two populations at baseline when all subjects were assessed using LCAS, which could bias the outcomes. Unidimensionality of LCAS was assessed through correlation between individual item scores with the total score. Internal consistency reliability was assessed using Cronbach's alpha. The dimensionality of the underlying subspace of latent variables (the dimensionality of the target construct) was assessed by factor analysis.

Although not previously used in psychometric scale development in veterinary behavioral medicine, interesting insights into the relevance and usefulness of single items can be drawn from item response theory (IRT). IRT is most widely used in psychometry to calibrate and evaluate items in tests, questionnaires, and other instruments. Nowadays, psychological and educational tests are built using IRT, because the methodology can significantly improve measurement accuracy and reliability while providing potential significant reductions in assessment time and effort. A unidimensional two-parameter model with logit link function (graded response model) was used for the evaluation of LCAS.

Finally, the practical-related aspect of LCAS validation required demonstration of treatment sensitivity (differentiation between treatment and placebo groups), sensitivity to the level of the invoking stimulus (response score within the placebo group around the time of peak challenge), and a correlation to other respondents' score (owner satisfaction score). To do this we examined the performance of the instrument in the relevant aspects indicated in the aforementioned noise fear study (15).

## Results

### Demographic Characteristics

The 226 dogs in the noise fear study (15) were an average median of 7 years old (range 1–14 years) and more than half of the patients were female (60.2% females; 39.8% males). The majority of dogs (81.4%) were neutered or spayed. There was no substantial overrepresentation of certain breeds apparent, and most dogs were mixed breeds (40.7%). Body weight was on average 20.0 kg (SD 12.9; range 3–72 kg). In 22.6% of cases at least one pre-existing condition was reported, which covered a variety of diseases. No disease category was observed in more than 10% of patients. Hypothyroidism was more frequently represented in the placebo (7.4%) than the imepitoin treated patients (3.8%). Most dogs did not show abnormalities of clinical concern in the physical examination at inclusion.

All included dogs were diagnosed with Noise Phobia, on the basis of medical history and applying the Lincoln Sound Sensitivity Scale as described above. The median value in the Lincoln Sound Sensitivity Score at screening was 75.5 (range 32–155). The minimum number of signs shown by any dog recruited in this study was 4 (mode = 9 signs). The most common sign reported at inclusion (i.e., prevalence with score > 0) was shaking or trembling (95.1%), followed by cowering (94.2%), hiding (93.8%), bolts (87.2%), and restlessness/pacing (85.5%). Many of these signs are clearly indicative of a response interfering with the dog's ability to function effectively in the home (a classic feature of mental illness in people). Overall, 85% of dogs were reported with at least one sign at its highest severity (i.e., frequency score of 3 x intensity score of 5 = total score of 15).

Overall, the demographic data and baseline characteristics were well-balanced between the Placebo and Imepitoin treatment groups (15).

### Construct Validity of the Scale

#### Face Validity

The behaviors used LCAS are a widely accepted representation of canine anxiety-related behaviors (16, 21–23).

The scale consists of 5 severity grades in accordance with the generally perceived good potential reliability and validity of 5–7 point scales (24).

#### Internal Consistency

**Figure 2:**
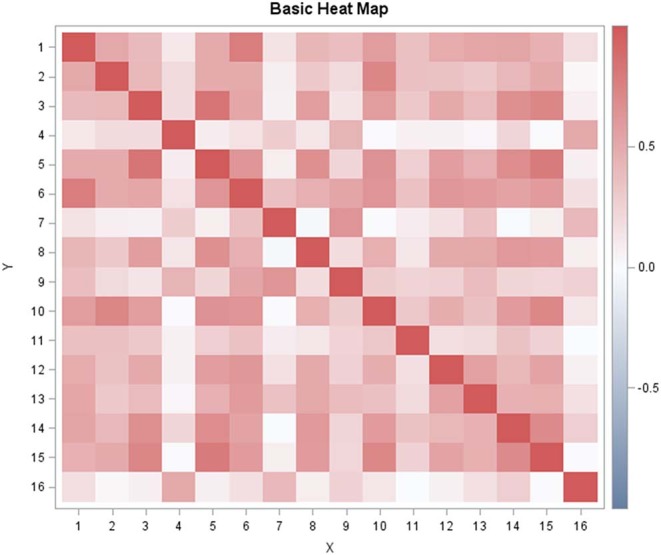
Visualization of the item-to-item correlation matrix (heat map) for Lincoln Canine Anxiety Scale (LCAS). The following item codes were used: 1 = running around; 2 = drooling salvia;, 3 = hiding; 4 = destructiveness; 5 = cowering; 6 = restlessness/pacing; 7 = aggressive behavior; 8= freezing to the spot; 9 = barking/whining/howling; 10 = panting; 11 = vomiting/defecating/urinating/diarrhea; 12 = owner seeking behavior; 13 = vigilance/scanning of the environment; 14 = bolts; 15 = shaking/trembling; 16 = self-harm.

Pairwise correlations between the single item scores of all dogs at baseline are shown in the heat plot (Figure 2), with positive correlation coefficients colored red, negative values blue and zero correlations white. This clearly shows that “destructiveness,” “aggressive behavior,” “barking/whining/howling,” “vomiting/defecating/urinating/ diarrhea,” and “self-harm” only loosely correlate with the remaining items. For these 5 weakly correlating items, correlations with the total sum score are 0.05, 0.11, 0.27, 0.2, and 0.06, respectively. For all other items, correlations with the total are >0.48, with six items correlating more than 0.65. Cronbach's alpha coefficient is α= 0.88, indicating good internal consistency. Removing the 5 weakly correlating items from the sum score produced only a small increase in α.

**Figure 3:**
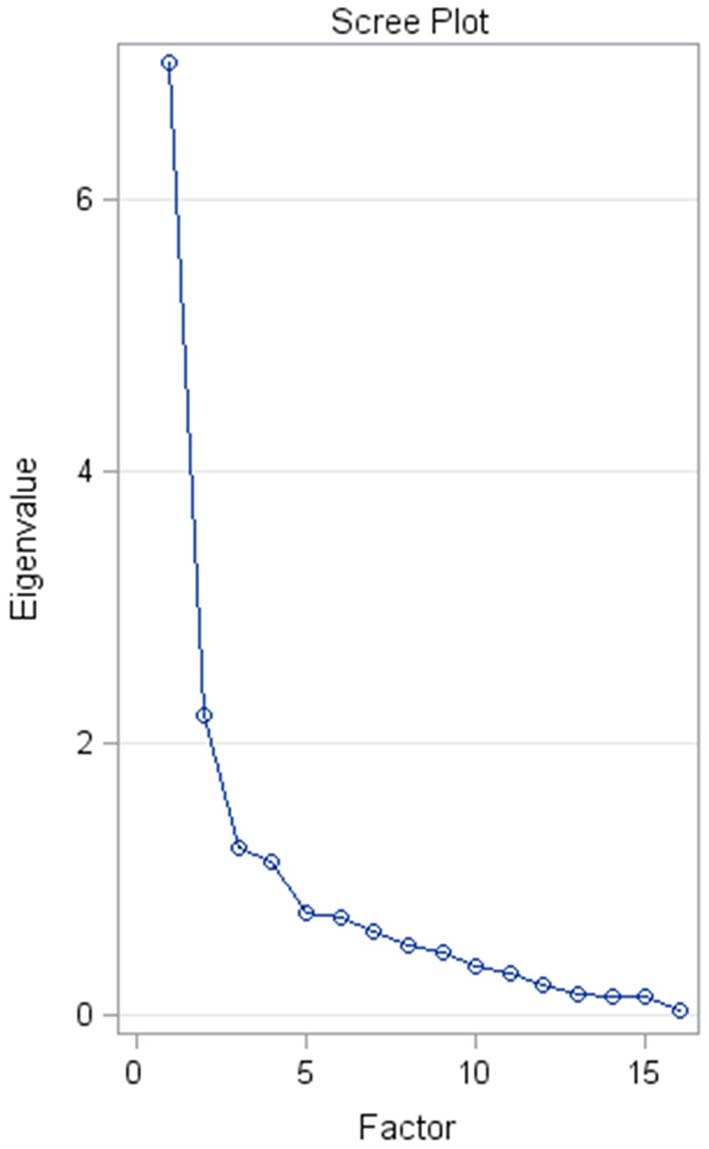
Scree plot of the eigenvalue spectrum of the correlation matrix for The Lincoln Canine Anxiety Scale (LCAS). The x-axis shows the index of the factor (= eigenvector), while the y-axis indicates the size of the respective eigenvalue. The first factor largely dominates the eigenvalue spectrum.

A principal component analysis (PCA) of the correlation matrix reveals that the eigenvalue spectrum is dominated by the first eigenvalue (see scree plot in Figure 3) with a clear inflection to the succeeding ones. This supports the fundamental underlying assumption that the sum score is largely explained by a single factor: the latent construct of “dog anxiety.”

**Figure 4:**
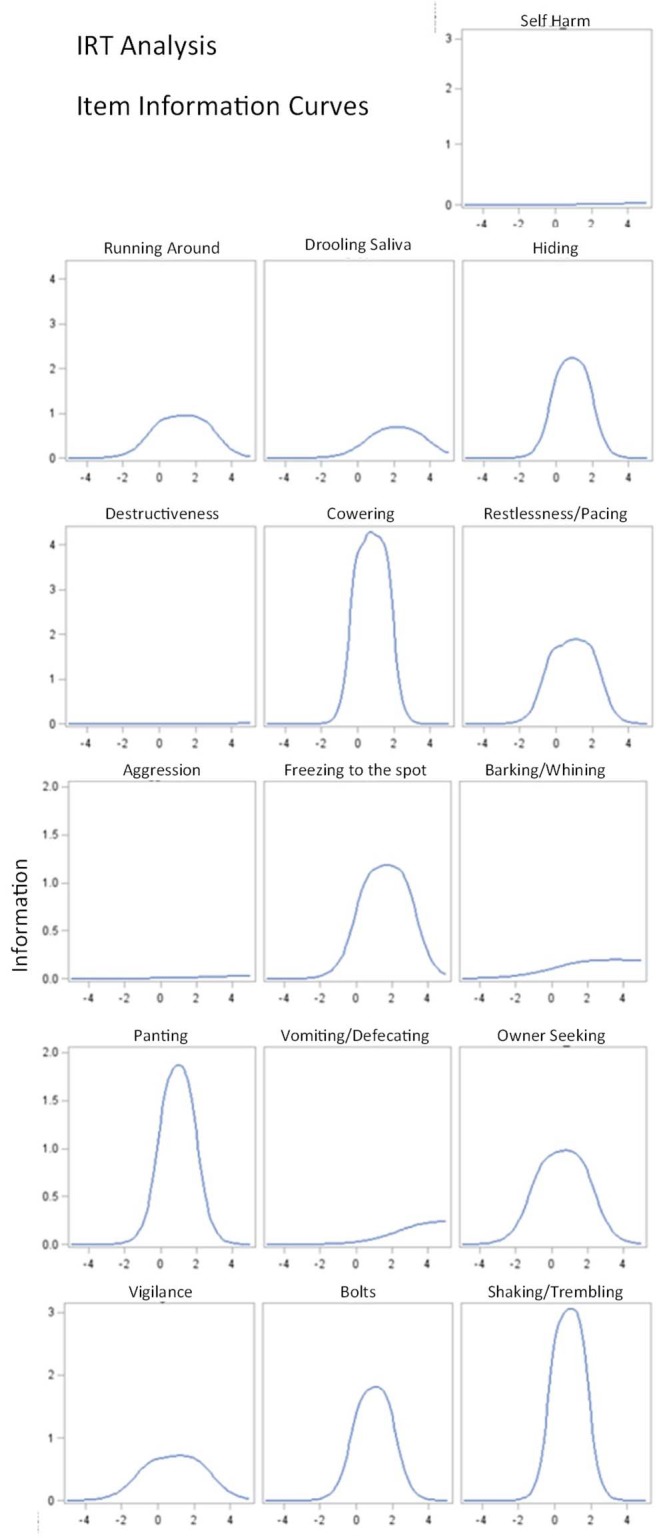
Item information curves for the Lincoln Canine Anxiety Scale (LCAS). The x-axis measures the size of the latent trait (here the anxiety level) in arbitrary units. The Y–axis is the Item Information Function (IIF), indicating where an item is most informative w.r.t. the latent trait. The steepness and sharpness of the curve reflect the discriminating power of the respective item and thus the contribution the item makes to the precision of the measurement. Items with steeper curves are more useful.

Further insight into the validity of the score was gained from the application of IRT. The resulting item information curves are drawn as a function of the latent trait (= anxiety level; Figure 4). Item information curves indicate where an item is most informative depending on the level of a latent trait. Items with high, sharp maxima are more informative than those that are flat or with a low slope. The panel of curves effectively visualizes the previous result indicating that the items “destructiveness,” “aggressive behavior,” “barking/whining/howling,” “vomiting/defecating/urinating/diarrhea,” and “self-harm” do not noticeably contribute to the total test information. Though these items might be crucial to fully describing all aspects of a dog's anxiety that may be of concern to an owner, these behaviors turn out not to be instrumental in this study. These items could be dropped from the scale without loss of important test information.

### Anxiety Scale Distribution Characteristics

To characterize the score characteristics in an anxiety-eliciting context we analyzed the behaviors in the subgroup of 120 placebo treated dogs assessed at 0:20 h, the time point of maximum noise stimulus during New Year's Eve fireworks.

Most owners observed cowering (87.5%), hiding (85%), shaking or trembling (84%) or owner seeking behavior (82%) in their dogs at the time of sampling during the noise event. Aggressive behavior (6%), self-harm (5%), vomiting/defecating/urinating/diarrhea (4%), and destructiveness (1%) were least frequently reported in response to noises (Table 4).

**Table 4:** Behaviors observed in placebo treated animals during a fireworks event (i.e., time point 0:20 h).

**Behavior**	**All Placebo treated dogs**			**Gender**		**Reproductive status**	
	**Total**	**No of dogs**	**Fraction (%)**	**Male (%)**	**Female (%)**	**Intact (%)**	**Neutered/spayed (%)**
Cowering	120	105	87.50	91.30	85.14	86.36	87.76
Hiding	120	102	85.00	84.78	85.14	90.91	83.67
Shaking or trembling	120	101	84.17	82.61	85.14	86.36	83.67
Owner seeking behavior	120	98	81.67	84.78	79.73	77.27	82.65
Restlessness/Pacing	120	92	76.67	82.61	72.97	77.27	76.53
Vigilance scanning of the environment	120	84	70.00	67.39	71.62	63.64	71.43
Bolts	120	81	67.50	60.87	71.62	63.64	68.37
Panting	120	81	67.50	71.74	64.86	59.09	69.39
Running around	120	79	65.83	65.22	66.22	59.09	67.35
Freezing to the spot	120	58	48.33	52.17	45.95	45.45	48.98
Drooling Saliva	120	49	40.83	45.65	37.84	50.00	38.78
Barking/Whining/Howling	120	33	27.50	28.26	27.03	13.64	30.61
Aggressive behavior	120	7	5.83	6.52	5.41	0.00	7.14
Self-harm	120	6	5.00	2.17	6.76	4.55	5.10
Vomiting, Defecating, Urinating, and/or Diarrhea	120	5	4.17	2.17	5.41	0.00	5.10
Destructiveness	120	1	0.83	0.00	1.35	0.00	1.02

*Percentages are based on observations of 120 animals (74 female/46 male; 22 intact/98 neutered or spayed). The behaviors of the score are sorted in descending order by frequency. Animals expressed frequently more than one behavior*.

**Figure 5:**
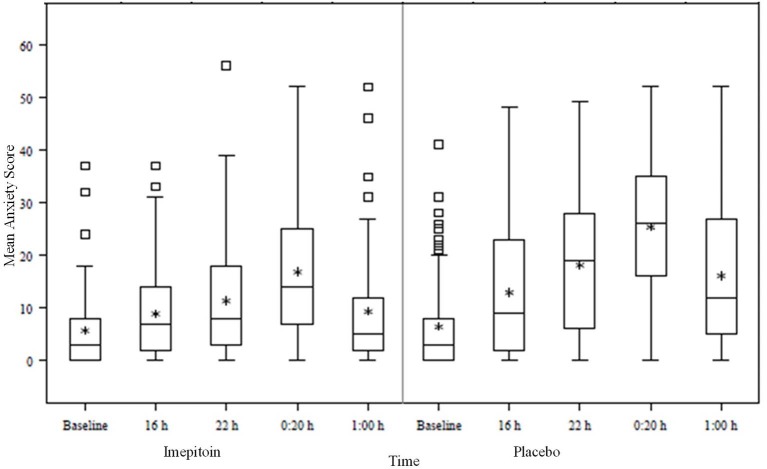
Box plot of the time evolution of the Lincoln Canine Anxiety Scale (LCAS—sum score) during New Year‘s Eve. The baseline value was recorded 5 days before the noise event.

In relation to total sum scores, owners reported a median score of 25.0 points (range 0–55; mean 24.9, SD 13.1) for placebo treated dogs during the fireworks event (time point 0:20 h in Figure 5). There was no relevant difference with respect to either gender (male 24.1 SD 11.1; female 26.1 SD 11.3) or combined neuter-gender status (male intact 25.7 SD 11.8; male neutered 26.3 SD 11.3; female intact 25.7 SD 17.4; female spayed 23.8 SD 13.6).

With the exception of barking/whining/howling, there were no relevant differences in specific observed behaviors evident with respect to gender or reproductive status. For barking/whining/howling the observed frequency was more than double in the spayed/neutered dogs compared to reproductively intact ones.

### Predictive Validity

#### Practice-Related Validation (Treatment and Stimulus Sensitivity)

An important feature of a clinical rating scale is the ability to reliably measure treatment effects. For this, the comparison between treatment groups in the clinical trial on the efficacy of imepitoin compared to placebo to alleviate anxiety and fear in response to firework noises on New Year's eve (15) was used. This study found that owners were around 4.7 times more likely to report a favorable response in their dog when using the medication compared to placebo. The last 4 time points assessed using LCAS were recorded during New Year's Eve, while the baseline value was taken a couple of days beforehand when the dog was in a calm state. It is clear (15) that the instrument is able (i) to discriminate between the treatment groups (sum score levels for placebo are higher than for active = Imepitoin) and (ii) to resolve the time evolution of the animals' anxiety level, which is in line with the typical course of the noise stimulus during New Year's Eve; indeed in the period just after midnight on New Year's day (the time for the highest intensity of fireworks), scores increased compared to baseline by an average of 24.9 +/– 13.1 (mean +/– standard deviation) in the placebo group, but by only 16.6 +/– 11.6 in the imepitoin treated group.

#### Respondent Related Validation

**Figure 6:**
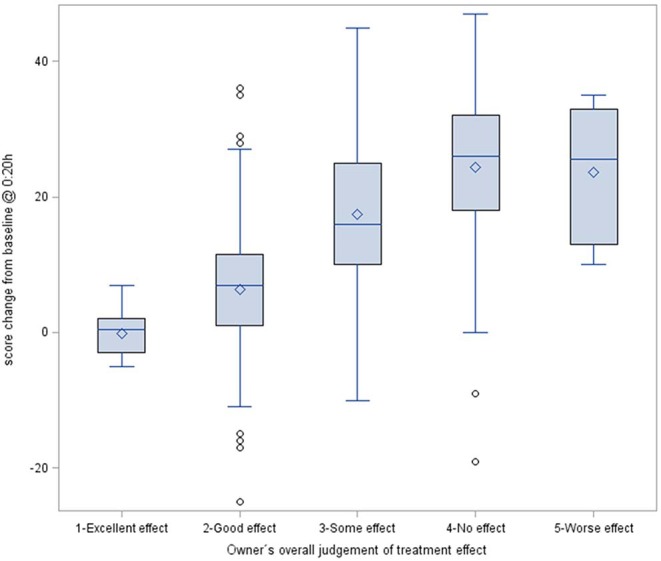
Box plot of the score change from baseline data compared to fireworks on New Year's Eve at 0:20 h (imepitoin and placebo group combined). The difference in score was plotted against the owner's proxy assessment of overall treatment effects (Table 3).

The owner's overall assessment of the treatment effect measured on a 5-grade ordinal scale [2nd co-primary endpoint of the noise fear study (15)] is a supplementary tool, which was used to check for the validity of the score (Figure 6). To that end the sum score change relative to baseline recorded at 0:20 h (at the peak of the noise stimulus) was correlated with the owner's subjective rating of the treatment effect [see (15) for further details]. The resulting Spearman correlation coefficient was 0.64 (*p* < 0.0001) indicating a good association between observed score change and owner's overall treatment assessment.

#### Treatment Sensitivity and Clinically Relevant Improvements

To assess the clinical relevance of the treatment effect, it is informative to relate the magnitude of the observed sum score change to the owner's overall perception of the treatment success. The plot indicates that a score change of roughly 20 points makes the difference between “no/worse” effect and an “excellent” effect. In other words, this provides the benchmark for assessing treatment improvement. For the noise fear study the observed treatment effect was a mean reduction by 6.1 scoring points (active vs. placebo). In comparison to the above benchmark, this a substantial (= clinical relevant) 30% difference.

## Discussion

In this paper, we not only validate a scale to assess canine anxiety, but also undertake several analyses relating to IRT on the properties of the scale, which have not been used before in the development of a psychometric scale within veterinary behavioral medicine. The scale also provides a benchmark for assessing a clinically valuable reduction in anxiety-related signs in future studies. Data were taken from the assessments of 226 patients in a controlled clinical trial for noise phobia conducted under good clinical practice guidelines. These were used to examine distribution characteristics, internal consistency, validity, and treatment sensitivity. Our findings provide strong support for the validity of this anxiety scale for dogs. Given the generalizability of these signs to a range of problems and disorders in dogs (20, 23), we suggest that this instrument, can be used in a much wider range of contexts.

Criterion validity cannot be assessed, as there is no gold standard available, and also a comparison to other independent and indisputable clinical signs is not useful due to the nature of behavior responses in this sort of condition or similar ones without an absolute biomarker like “pain” or “quality of life.” For such abstract constructs, validation of a measurement involves a series of steps known as “construct validation” (11). All our results support the fundamental underlying assumption that the sum score is largely explained by a single factor, the latent construct “dog's anxiety.” Convergent validity of the canine anxiety score was derived from correlation with the owner's assessment of overall treatment effects.

We have demonstrated the ability of this scale to reliably measure treatment effects. Dogs with noise phobia had a mean score of 24.8 points under placebo treatment, while imepitoin treated dogs reached in mean a score of 16.6 points during fireworks. Interestingly, the Hamilton Rating Scale for Anxiety (HAM-A) which is widely used to measure anxiety in a wide range of clinical studies (7), defines signs of mild anxiety as scores below 17, with scores above 25 indicating severe anxiety. In this scale for people, a reduction to 66% of the severe anxiety value represents the change to mild anxiety. By comparison, in this scale for dogs, we observed that a 30% reduction in anxiety score under treatment also appears to be a clinically relevant effect based on the good association between observed score change and owner's overall treatment assessment.

While our findings are highly supportive of the reliability and validity of the canine anxiety scale, it is important to note some current limitations. Firstly, the validation of this scale was performed only on the dataset of one study. While this was a large and well-controlled study conducted under good clinical practice guidelines, variability between different study setups are not reflected here. Secondly, the dataset consisted only of dogs with noise phobia and fireworks as eliciting context. The results resemble findings previously reported using this scale in a wider range of anxiety-related conditions (20). We therefore encourage clinicians and researchers to use this scale in a wider range of contexts involving canine anxiety, in order to determine its robustness more fully. Nonetheless, for now, this instrument provides the most robust way to assess canine anxiety. A short form that excludes the items “destructiveness,” “aggressive behavior,” “barking/whining/howling,” vomiting/defecating/urinating/diarrhea,” and “self-harm,” may also be used if none of these signs are a focus of the owner complaint.

## Conclusions

Through the application of item response theory alongside well-established methods of psychometric validation we have not only demonstrated that the Lincoln Canine Anxiety Scale is a reliable and valid measure of anxiety in dogs, but also developed a short form, for clinical use.

## Data Availability Statement

The raw data supporting the conclusions of this article will be made available by the authors, without undue reservation, to any qualified researcher.

## Ethics Statement

This study is a retrospective analysis of an existing data set, so no animals were included for this study. The original clinical trial (publication accepted by Journal of Veterinary Internal Medicine) had a different purpose and was carried out in accordance with the principles of the Basel Declaration and was approved prior to study start by the relevant authorities in Germany and the Netherlands. Ethical or IACUC approval are not necessary for data analysis of existing data sets, and this approach is encouraged by the 3R principle.

## Author Contributions

DM and KM originally developed the anxiety scale. DM, KM, and OE contributed to the analysis of the results and the creation of the final manuscript. HM performed the statistical analysis. OE performed the study to generate the data set with support from DM and HM. All authors had access to all study information and raw data, and approved the manuscript.

### Conflict of Interest

This publication followed the GPP3 guidelines. OE and HM are employees of Boehringer Ingelheim, Germany, the marketing authorization holder of Pexion^®^, containing Imepitoin as active principle. DM acted as consultant for Boehringer Ingelheim, and KM was involved in research sponsored by Boehringer Ingelheim.

## Abbreviations

IRT: Item Response Theory
SD: Standard Deviation.
